# A Systematic Review of Chest Imaging Findings in Long COVID Patients

**DOI:** 10.3390/jpm13020282

**Published:** 2023-02-01

**Authors:** Somayeh Bazdar, Anastasia K. A. L. Kwee, Laura Houweling, Yolanda de Wit-van Wijck, Firdaus A. A. Mohamed Hoesein, George S. Downward, Esther J. Nossent, Anke H. Maitland-van der Zee

**Affiliations:** 1 Department of Pulmonary Medicine, Amsterdam UMC, 1105 AZ Amsterdam, The Netherlands; 2 Amsterdam Institute for Infection and Immunity, 1105 AZ Amsterdam, The Netherlands; 3 Amsterdam Public Health, 1105 AZ Amsterdam, The Netherlands; 4 Department of Radiology, University Medical Center Utrecht and Utrecht University, 3584 CX Utrecht, The Netherlands; 5 Department of Environmental Epidemiology, Institute for Risk Assessment Sciences (IRAS), 3584 CX Utrecht, The Netherlands; 6 Julius Center for Health Sciences and Primary Care, University Medical Center Utrecht, 3584 CX Utrecht, The Netherlands

**Keywords:** long COVID, lung, chest, imaging

## Abstract

Long COVID is the persistence of one or more COVID-19 symptoms after the initial viral infection, and there is evidence supporting its association with lung damage. In this systematic review, we provide an overview of lung imaging and its findings in long COVID patients. A PubMed search was performed on 29 September 2021, for English language studies in which lung imaging was performed in adults suffering from long COVID. Two independent researchers extracted the data. Our search identified 3130 articles, of which 31, representing the imaging findings of 342 long COVID patients, were retained. The most common imaging modality used was computed tomography (CT) (N = 249). A total of 29 different imaging findings were reported, which were broadly categorized into interstitial (fibrotic), pleural, airway, and other parenchymal abnormalities. A direct comparison between cases, in terms of residual lesions, was available for 148 patients, of whom 66 (44.6%) had normal CT findings. Although respiratory symptoms belong to the most common symptoms in long COVID patients, this is not necessarily linked to radiologically detectable lung damage. Therefore, more research is needed on the role of the various types of lung (and other organ) damage which may or may not occur in long COVID.

## 1. Introduction

The worldwide crisis of Coronavirus disease 2019 (COVID-19) pandemic caused by the severe acute respiratory syndrome, coronavirus 2 (SARS-CoV-2), has influenced people’s lives globally [1,2]. On 30 January 2020, the WHO declared the outbreak of SARS-CoV-2 a Public Health Emergency of International Concern and, since then, COVID-19 has caused (as of 23 December 2022) at least 6,651,415 deaths and 650,879,143 confirmed cases. [3,4]

Although COVID-19 is primarily a respiratory disease, it may affect extra-pulmonary organs and cause systemic complications [5,6]. These complications can occur not just in the acute phase but also after the initial infection has been overcome [7]. These long-term (or chronic) complications are collectively described as “long COVID” and represent a variety of complaints. Presently, long COVID is defined as the persistence of one or more COVID-19 symptoms weeks or months after the initial viral infection [8,9]. Long COVID is probably one of the most important complications of SARS-CoV-2 infection; it can affect both the mental and physical ability of patients and have significant effects on society’s work force [10,11].

Although the etiology of long COVID is currently unknown, several studies have indicated that lung damage may play a significant role in its development [12]. This is supported by reports that the most common symptoms among long COVID patients are respiratory in nature [13]. During the initial phase of COVID-19, lung abnormalities are related to the course and prognosis of the disease. In the initial two weeks, the most common imaging finding is ground-glass opacity (GGO) followed by consolidation. From the second week of infection, however, imaging abnormalities suggestive of fibrosis might start to appear [14]. Radiological abnormalities can also be observed long after the initial infection has resolved. For example, CT scans of COVID-19 survivors who were admitted to the ICU during their acute phase showed that residual lung abnormalities can still be radiologically detected after three months. However, whether findings are clinically relevant has not yet been elucidated; these changes represent an ongoing recovery; thus, to distinguish them from irreversible fibrotic changes, further follow-up imaging after at least one year has been recommended [15].

Further evidence supporting the association between lung damage and the development of long COVID is detecting radiological abnormalities in patients who were infected with other coronaviruses (SARS and MERS) even years after infection. [16]. Serial lung high-resolution computed tomography (HRCT) scans of SARS-CoV-1 patients demonstrated that some patients still demonstrated fibrotic changes, and even ground-glass-like changes, in their long-term serial follow-ups after 12 months [17]. Moreover, in a one-year assessment of post-SARS-CoV-1 patients, a significant correlation between the percentage of lung imaging abnormalities and certain lung function parameters (specifically, the total lung capacity and diffusing capacity of the lungs for carbon monoxide) was reported [18].

The key question for patients and their pulmonologists is whether performing lung imaging in patients with long COVID is illuminative. As the COVID-19 pandemic has now been present for more than two years, sufficient time since initial infection has occurred to allow closer examinations of radiological changes in those with long COVID. However, there is presently no collective overview of the relationship between imaging findings and their outcomes. Therefore, the aim of this systematic review is to provide an overview of lung imaging findings in long COVID patients. 

## 2. Methods and Materials

This systematic review was conducted as per our registered protocol (PROSPERO Record ID = CRD42021292358) (https://www.crd.york.ac.uk/prospero/display_record.php?RecordID=292358, access on 26 November 2021) and findings are reported as per Preferred Reporting Items for Systematic Reviews and Meta-Analyses statement (PRISMA) guidelines. On 29 September 2021, we performed a PubMed search to identify articles examining lung imaging among adults suffering from long COVID. No explicit date was included in the search, although only studies published during the COVID-19 pandemic, from 30 January 2019, were considered. The search terms (see below) were selected based on the authors’ knowledge and on previously identified publications. Snowballing sampling was performed where the references of any identified review articles were also examined to identify any additional relevant articles.

*Search terms*: (((long COVID) OR (long COVID) OR (post acute sequelae of COVID) OR (pacs) OR (chronic COVID syndrome) OR (chronic COVID) OR (ccs) OR (long haul COVID) OR (long COVID haul*) OR (post COVID) OR (COVID surviv*) OR (late effect* COVID) OR (COVID late complication*) OR (post acute COVID) OR (long term COVID) OR (longterm COVID) OR (persist* COVID) OR (late COVID) OR (ongoing COVID) OR (Enduring COVID) OR (longlast* COVID) OR (lengthy COVID) OR (residual COVID) OR (relaps* COVID) OR (COVID remission*) OR (linger* COVID) OR (permanent* COVID) OR (subacute COVID) OR (sub acute COVID) OR (permanent* COVID)) AND ((chest) OR (lung) OR (thorax) OR (thorac*) OR (pulm*)) AND ((imag*) OR (radiolog*) OR (graph*) OR (scan) OR (computed tomography scan) OR (ct scan) OR (ct) OR (CAT) OR (computer assisted tomography) OR (hrct) OR (X ray) OR (cxr) OR (magnetic resonance imaging) OR (mri) OR (ultrasonography) OR (us)))

*Inclusion criteria*: English language studies in which any modality of lung imaging was performed on adults suffering from long COVID were eligible for inclusion, regardless of whether lung imaging was the only intervention in that study or one among several interventions. Preprints and letters to the editor were not considered valid for inclusion, nor were papers in which we were unable to distinguish between asymptomatic post-COVID patients and those with long COVID symptoms. 

Long COVID was defined following the Centers for Disease Control and Prevention’s (CDC) definition as patients with a confirmed diagnosis of COVID-19 (whether based on PCR test, serology test, any other laboratory test, clinic-radiological diagnosis, history of close contact with proven case) who continue to experience COVID-19 symptoms for more than 28 days.

**Figure 1:**
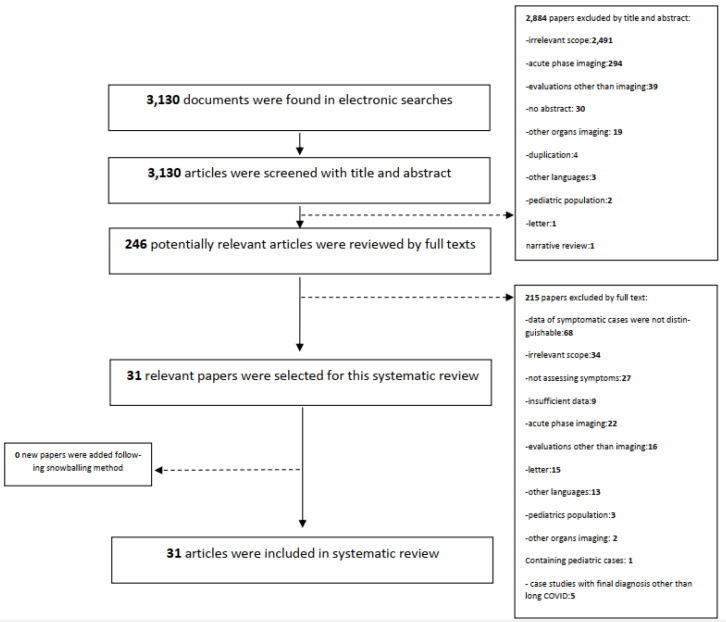
Flowchart of database search and article inclusion.

*Screening*: Title and abstract screening of the papers identified in our search was performed by SB. All articles identified through title and abstract underwent full text screening to ensure they matched our inclusion criteria prior to data extraction. As a quality control step, 10% of the identified articles (selected randomly) were evaluated by a second reviewer (AK). Disagreements in evaluations (which occurred three times) were discussed with a third reviewer (LH). All final determinations aligned with those of the main reviewer (SB). The flowchart of the screening process is provided in Figure 1.

*Data extraction*: Data from the identified articles were extracted into a customized spreadsheet containing study information (study design, publication year, number of participants, inclusion criteria, country and continent of the performed study, study period), demographic data (age, sex, ethnicity, nationality) and imaging/outcome information. Specific imaging and outcome information were as follows: percentage of long COVID cases with lung imaging abnormalities, the type of lung imaging abnormalities, lesion distribution, imaging interpreters, imaging modalities, imaging evaluation time, imaging evaluation setting, long COVID symptoms, medical interventions, probable risk factors for developing long COVID complications, such as smoking, obesity, comorbidities, and intubation. According to the anatomical positioning and physiology, we categorized the extracted imaging findings into the four groups: interstitial (fibrotic) abnormalities, other interstitial abnormalities, airway abnormalities, and parenchymal abnormalities. However, it should be noted that some specified abnormalities can fit into different categories.

*Risk of bias (quality) assessment*: The National Heart, Lung, and Blood Institute (NHLBI) risk of bias assessment tool was used to evaluate the risk of bias and was performed by two reviewers (SB, AK) independently. There was complete agreement between these reviewers regarding quality assessment.

## 3. Results

Our literature search identified a total of 3130 articles. After title and abstract screening, we removed 2884 papers, while a further 215 articles were removed after full text screening, leaving 31 articles for data extraction. Primary reasons for paper exclusion during the title and abstract screening stage were as follows: non-COVID-19 diseases, addressing the technical aspects of artificial intelligence usage in COVID-19 lung imaging, imaging findings being reported only from the acute phase of COVID-19, and non-lung imaging findings. During full text screening, several articles were excluded as data from symptomatic patients could not be distinguished from the data of asymptomatic ones (N = 68). Other reasons for exclusion during the full text screening step included: topics irrelevant to the aim and scope of our systematic review (N = 34), not assessing symptoms in the follow-up evaluation (N = 27), insufficient information about participants or intervention such as, participants age range, evaluation time, COVID-19 diagnosis method, etc., that caused them not to fulfill our inclusion criteria clearly (N = 9), and case-reports with final diagnoses other than long COVID (N = 5). The flowchart of the screening process, as well as the exclusion reasons for each step, is provided in Figure 1.

After title, abstract, and full-text screening, 31 articles were identified that met our inclusion criteria, representing the imaging findings of 342 patients who were suffering from long COVID. Of these 31 articles, 9 represented cohort/case-control studies, covering 309 participants; 5 were case series, covering 16 patients; and 17 were individual case reports. The study population tended to be male (62.2%) with a mean age of 59.5 years. The study design of the original studies included in this systematic review were either cohort or case-control (Table 1). With the exception from one case-report, the quality of all studies was fair based on the NHLBI checklist. More extensive information, including the outcome of quality assessment, is presented in the Supplement (Table S1).

The number of patients undergoing imaging ranged from four to 91 in the identified studies. Imaging was typically performed in one of three settings: follow-up evaluation, re-admission, and prolonged hospitalization. Case reports and series occurred exclusively in the re-admission and prolonged hospitalization setting whereas, with a single exception (Gaspardone et.al.) all of the original articles occurred in the follow-up evaluation setting.

Disease severity and/or used interventions were inconsistently reported across studies and not explicitly linked to radiological findings. For example, the intubation status of patients studied was not mentioned for 201 cases. In the remaining 141 cases, 63 were intubated and 78 were not (Table S2). While medication use was similarly inconsistently reported, among the medications that were used during the acute phase of COVID-19 infection, hydroxychloroquine was the most commonly used, followed by antiviral medications such as remdesivir, oral antibiotics such as azithromycin, and interlukin-1 (IL-1) inhibitors such as anakinra (Table S4). Among the 111 patients where comorbidities were reported, hypertension was the most common comorbidity (n = 61), followed by diabetes (n = 28) (Table S3).

Dyspnea was the most common Long-COVID symptom in patients, followed by fatigue. Supplement shows the frequency of all long COVID symptoms for each study (Table S4). 

Across all settings and study types, the most common imaging modality used was CT scan (N = 249), followed by USS (N = 73), CXR (N = 25), positron emission tomography (PET) scan (N = 23), and magnetic resonance imaging (MRI) (N = 2) (Table 1 and Table S5). Across these modalities, a total of 29 different imaging findings were reported, with a variety of reporting techniques and terminology. On the basis of anatomical positioning and physiology, we categorized these findings into the following groups: interstitial (fibrotic) abnormalities, pleural abnormalities, airway abnormalities, and other parenchymal abnormalities (Table 2). In two articles, the findings were reported as general terms of COVID pneumonia (Malik et al.) or bilateral pneumonia/viral pneumonitis (Mitrani et al.), and thus could not be categorized into any group. Several of the cohort/case-control articles (Armange et al., Bai et al., Cesarone et al., Rinaldo et al., Sollini et al., Sollini et al., Miwa et al.) also reported the number of long COVID patients without any residual lesions on their imaging. These articles represented 148 patients, of whom 82 (55.4%) had positive findings on their imaging (Table 3). All case studies and reports had positive imaging findings (Table S5). 

When categorizing specific radiological findings, other parenchymal abnormalities were the most reported finding (112 times), of which ground-glass opacity (reported 93 times) was the most common specific abnormality. The second most common abnormality grouping was interstitial (fibrotic) abnormalities, reported 105 times. Within this group, reticulation (reported 78 times) and fibrotic changes (reported 14 times) were the most common specific abnormalities. Airway and pleural abnormalities were reported relatively less than the other categories (28 and 7 times, respectively (Table 2)). Only two studies (Armange et al. and Yin et al.) assessed the relationship between symptoms and radiological findings, with contradictory findings. Armange et al. reported that there was no relationship between post-COVID symptoms and CT scan findings [19]; however, Yin et al. found that there was a relationship between post-COVID symptoms and imaging findings, and that dyspneic long COVID patients had larger lesions, a higher residual lesion rate, and incompletely absorbed lesions [26].

While none of the patients that were categorized into the re-admission and prolonged hospitalization groups had negative imaging findings, 46% of the patients evaluated in the outpatient follow-up setting had negative imaging findings (Table 4, Table S6). In the prolonged hospitalization group other parenchymal abnormalities were more frequent; while in the follow-up evaluation and re-admission groups, interstitial (fibrotic) abnormalities followed by other parenchymal abnormalities were the most frequent lung imaging abnormalities (Table 4). 

When examining only the cohort/case-control studies to assess their findings based on the evaluation setting, we found that their imaging was all performed in the follow-up setting, except for the study conducted by Gaspardo et al., whose patients were evaluated by LUSS in a prolonged hospitalization setting. Moreover, while Gaspardo et al. did not report on specific findings or on positive versus negative findings, they commented that the LUSS severity score at discharge was higher among those with worse disease at admission. Table 5 illustrates the imaging findings of the cohort/case-control studies, which were conducted in the follow-up setting. In these studies, the most reported finding was interstitial (fibrotic) abnormalities (58.2%), followed by other parenchymal abnormalities (53.8%) and airway abnormalities (13.9%). No pleural abnormalities were reported in these studies.

There were eight studies in this systematic review which compared lung imaging from two different modalities (Table 6). Sollini et al. compared CT vs. PET in two studies, with somewhat contradictory findings. In a study of ten patients, fibrotic changes were reported on CT for six of them, while only two cases had mild [18F]FDG lung uptake [24]. However, in an evaluation of a different group of thirteen patients, [18F]FDG uptake was detected in six patients, with bilateral findings being observed on CT for only four patients who only had mild [18F]FDG uptake [25].

Garg et al. and Heiss et al. both compared CT and MRI scans for single patients, reporting identical findings in both cases [28,29]. Tung Chen et al. compared USS to CT and found anatomically correlated radiological residuals in both imaging techniques; however, they could not specify the type of abnormality in USS [30]. Finally, four papers [31,32,33,34] compared plain CXR findings to CT scan, finding that while radiological residuals were visible on CXR, they lacked the more precise definition of CT scan.

**Table 6:** Comparison of various imaging modalities.

First Author	Compared Modalities and Performing Days *	Imaging Modality	Imaging Finding
Sollini et al. [24]	30 days	CT	Fibrotic change in 6 cases out of 10 population of study
		PET	[18F]FDG lung uptake in 2 cases out of total 10 population of study
Sollini et al. [25]	132 days	CT	bilateral lung abnormalities, as typically observed in recovered COVID-19 pneumonia, was detected in 4 cases out of 13 in the total population of study
		PET	Mild [18F]FDG PET/CT uptake was detected in of Post-pneumonia lung abnormalities in 4 cases out of 13in the total population of study, and 2 other patients presented moderate/high [18F]FDG uptake in the lung, mediastinal lymph nodes, soft tissue, and breast tissue related to their comorbidities.
Garg et al. [28]	CT at 53 days and MRI at 55 days	CT	interlobular septal thickening, parenchymal band, Fibrotic change, bronchiectasis, bronchiolectasis, crazy-paving pattern
Garg et al. [28]		MRI	interlobular septal thickening, parenchymal band, Fibrotic change, bronchiectasis, bronchiolectasis, crazy-paving pattern
Heiss et al. [29]	97 days	CT	GGO, Consolidation
Heiss et al. [29]		MRI	GGO, Consolidation
Tung-Chen et al. [30]	56 days	CT	pleural thickening
Tung-Chen et al. [30]		USS	irregular pleural line in the right lateral area of the chest, which correlated with an area of pleural thickening on chest CT
Tung-Chen et al. [30]		CT	GGO
Tung-Chen et al. [30]		USS	a mild irregular pleural line and B-lines in the right anterior area of the chest, which correlated with ground-glass opacities on chest CT with no abnormal findings in the pleura.
Tung-Chen et al. [30]		CT	fibrotic change
Tung-Chen et al. [30]		USS	a marked irregular pleural line and multiple B lines, especially in the posterior inferior area of the chest, which correlated with fibrotic changes on chest CT
Alhiyari et al. [32]	130 days	CXR	air space opacity
Alhiyari et al. [32]		CT	GGO, bronchiectasis, interlobular septal thickening, reticulation, honeycomb-like appearance, interstitial pneumonia pattern
Hamad et al. [33]	CT at 35 days and CXR at 36 days	CXR	pneumothorax, air space opacity
Hamad et al. [33]		CT	pleural effusion, atelectasis, pulmonary edema, cystic airspace
Aesif et al. [31]	CT at 59 days and CXR at 122 days	CT	pneumothorax, Consolidation
Aesif et al. [31]		CXR	complete opacification, volume loss
Malik et al. [34]	42 days	CXR	Consolidation
Malik et al. [34]		CT	GGO, Fibrotic change, bronchiectasis, architectural distortion, linear scaring, airspace disease, COVID pneumonia

Footnote: * The performance day refers to the number of days that have passed since the onset of symptoms and in which the imaging evaluation was performed.

Regarding the imaging interpreter, there were only three studies, performed by Sollini et al. and Yin et al. [23,24,26], which mentioned the interpretation procedure and specialist who reported the imaging findings. In both studies of Sollini et al., it was noted that interpretation was performed by nuclear medicine physicians, and details about the experience of the interpreter or any available guidelines that they might have used were not reported. Yin et al. reported that two radiologists with 15 and 10 years of experience in cardiothoracic imaging reported the radiologic imaging. However, information related to the guidelines on which they based their interpretations and reports was not included.

## 4. Discussion

The role of lung imaging in the care of long COVID patients is an area of uncertainty for physicians and patients. While physicians should consider precise evaluation of the respiratory system through imaging, exposing patients to unnecessary doses of radiation and straining health care resources should be also avoided. Therefore, obtaining a comprehensive insight, including indications for lung imaging in long COVID patients, is essential. This systematic review aimed to develop an understanding of the current role of imaging (and its findings) among those with long COVID.

A major finding of this systematic review was that although the dominant symptoms in long COVID patients are respiratory, this is not necessarily related to lung imaging abnormalities. Overall, approximately 60% of patients included in the cohort and case-control studies in this review had radiological residuals, the most common of which was parenchymal abnormalities other than interstitial (fibrotic) abnormalities. Moreover, the findings of the only two studies assessing the relationship between the findings and the extent of symptoms [20,26] were not in accordance with each other. Together, this indicates that chest imaging alone is not sufficient for the evaluation of long COVID, and that it should instead form one part of overall patient care.

This review identified that CT scans were the most commonly used modality for imaging in long COVID. However, there were studies using modalities other than CT scans, such as CXR, US sonography, MRI, and even PET scans in long COVID patients. These imaging techniques are quite novel, unusual, and clinically irrelevant in this area, and in clinical practice setting, the common imaging technique was firstly the CT scan and secondly CXR. Almost all of the studies using modalities other than the CT scan and CXT were performed in an experimental setting, while the studies employing CT scans and CXR as imaging modalities were mostly conducted in a setting of routine follow-up evaluation of patients.

Studies comparing MRI versus CT [28,29] reported consistent findings between the two. However, since the identified articles only covered the findings from two patients, further investigations in larger scale settings are needed to better understand the relative role of these imaging modalities. In routine medical practice, CT is generally considered superior to MRI for imaging of areas prone to movement, such as the lung [35]. It should also be considered that MRI is the more expensive and time-consuming modality, and any potential benefits need to be weighed against the higher costs and lower availability.

US sonography is mostly used in the acute phase, and especially for obstetric or pediatric patients [36,37]. However, Gaspardone et al. and Tung-Chen et al. found that this modality would also be helpful in detecting the long-term chest findings of COVID-19 patients [22,30]. While US sonography is among the safest imaging modalities since it is a radiation-free technique [38], the precision of this modality, especially for the respiratory system, will never be as high as a CT scan, and the detailed evaluation of parenchymal is necessary for patients with prolonged respiratory symptoms. Therefore, some believe that the clinical application of this technique in long COVID is to be the first-line assessment and guide the physicians to perform CT scans for cases that had USS findings [39]. Furthermore, since US sonography is an operator-based modality [40], providing a standard protocol for post-COVID US sonography is also necessary. 

The PET scan was utilized in two studies of this systematic review [24,25]. This modality is typically used for detecting areas of activity in the body, such as cancer and inflammation [41]. However, the role of this method in lung evaluation of long COVID patients is less clear, since in some abnormalities no active inflammation is happening (e.g., a fibrotic lesion) and cannot thus be detected in this way [42]. Moreover, pre-existing disease or comorbidities may result in [18F]FDG uptake, causing a false positive finding. Despite these limitations, this imaging technique may be appropriate in terms of searching for the systemic etiology of long COVID, as well as tracking the treatment response to the anti-inflammatory medications that has been administrated for long COVID patients with lung inflammation. 

Comparing the imaging findings that were reported through CT scan and CXR showed that the abnormalities can be reported more precisely through CT scan compared with CXR. This may indicate that CXR is a sub-optimal modality for providing detailed lung evaluation in long COVID cases. However, this low-dose radiation lung imaging technique [43] can be a screening method among long COVID patients to determine the proper candidates for a CT scan.

Air trapping, identified as a diffuse lung injury that might be developed by the inflammatory obstruction of small airways [44], was not reported in any of included articles. Neither was it reported whether it was even examined during the imaging. This finding is a sign of the obstructive problems in small air ways and has been reported in long COVID patients [45,46]. Therefore, this radiological finding should be examined in long COVID lung imaging. 

COVID-19, and by extension long COVID, are novel diseases, meaning that the ability of imaging devices and interpreters to accurately identify relevant findings is more limited than for other diseases. Using either a computer-based or a human-based method to determine the findings affects the precision of the imaging reports. In human-based reports, the expertise and experience of the interpreter are important, an importance which is likely to be increased in the context of a new and emerging condition [47,48]. Although one of our aims was to evaluate what the imaging interpretations method is, only three studies [23,24,26] mentioned this point. 

An additional challenge is that while we sought to examine long COVID, this condition was rarely (if ever) being explicitly examined. Most of the available articles were assessing all post-COVID patients, and, where possible, we focused on only symptomatic cases, extracting information specifically related to long COVID. This highlights a need for research more specifically focusing on long COVID. Another limitation of this study is that there are many different radiological terms, such as "interstitial pneumonia pattern” and “volume loss of lung” used in the included article, for which there are no clear definitions but some potential overlap in what the term means, which allow some specified abnormalities to fit into different categories.

## 5. Conclusions

In this review of imaging studies, we identified a variety of radiological findings among patients with long COVID, including scans where no abnormalities were found. This indicates that in order to properly evaluate long COVID, a holistic view is required, which will consider both the individual patient and the potential role of other organs and systems.

The small number of included articles indicates that lung imaging findings in long COVID patients is an area that requires more research.

Considering the burden of long COVID syndrome and the remaining uncertainties, more research which specifically examines long COVID is urgently required. Furthermore, the correlation between the imaging findings of long COVID patients and their underlying diseases, predisposing factors, medications that were consumed during the acute phase, and their symptoms should be studied.

## Figures and Tables

**Table 1:** Details of the original studies in this systematic review.

First Author/Group of Articles	All Study Participants	Long-COVID Patients Who Underwent Imaging	Imaging Modality	Mean Age	Male	Study Design
Original articles	761	309	CT, USS, CXR, MRI	59.7	147 (61%)	Cohort/Case-control
Armange et al. [19]	214	23	CT	44	5(21.7%)	Cohort
Bai et al. [20]	7	4	CT	62.75	2 (50%)	Case-control
Cesarone et al. [21]	18	18	CXR	56.9	10(55.5%)	Cohort
Gaspardone et al. [22]	70	70	USS	68	48 (68.5%)	Cohort
Rinaldo et al. [23]	75	68	CT	NM	NM	Cohort
Sollini et al. [24]	101	10	CT & PET	58	7 (70%)	Cohort
Sollini et al. [25]	13	13	CT & PET	54	8 (61.5%)	Case-control
Yin et al. [26]	337	91	CT	58.68	57 (62.6%)	Cohort
Miwa et al. [27] *	17	12	CT	63.25	10 (83.3%)	Cohort
Case reports/series	33	33	CT, USS, CXR	57.5	23 (71%)	Case report/case series

Abbreviations: CT; Computed Tomography, CXR; Chest X-Ray, PET; Positron Emission Tomography, MRI; Magnetic Resonance Imaging. NM; Not Mentioned, US; Ultra-Sound Sonography. *: For this study the median for age was available and inserted here instead of the mean.

**Table 2:** Frequency of radiological findings in long COVID patients and their categorization.

Category of Abnormality	Frequency (N)	Specified Abnormality	Frequency (N)
Interstitial (fibrotic) abnormalities	105	Reticulation	78
		Architectural distortion	2
		Honeycomb-like appearance	2
		Linear scarring	1
		Fibrotic change	14
		Parenchymal band	2
		Fibrous stripe	3
		Scissural deformation	1
		Interstitial thickening	2
		Interlobular septal thickening	2
		Pulmonary edema	8
		Interstitial pneumonia pattern	1
Pleural abnormalities	7	Pneumothorax	4
		Pleural effusion	6
		Hemopneumothorax	1
		Pleural thickening	1
Airway abnormalities	28	Bronchiectasis	4
		Bronchiolectasis	25
Other parenchymal abnormalities	112	Ground-glass opacity	93
		Consolidation	22
		Complete opacification	1
		Airspace opacity	4
		Reticular opacity	5
		Aeriation	1
		Volume loss of lung lobe	7
		Emphysematous changes	4
		Atelectasis	1
		Crazy paving pattern	16

Footnote: most of the patients had multiple specified abnormalities and all of them belonged to one of the categories; hence, the sum of the specified abnormalities for each category is greater than the frequency of that category.

**Table 3:** Imaging modalities and imaging findings of the included case-control and cohort studies.

First Author	Evaluation Setting	Evaluation Time (days)	Imaging Modality	Imaging Finding	Positive FU Imaging	Negative FU Imaging	Category Interstitial (Fibrotic)	Category Pleural	Category Airway	Category Other Parenchymal
Armange et al. [19]	Follow-up Assessment	42	CT	GGO	4	19	0	0	0	4
Bai et al. [20]	Follow-up assessment	40	CT	Consolidation, fibrous stripe	4	0	3	0	0	1
Cesarone et al. [21]	Follow-up assessment	60	CXR	pulmonary edema	7	11	7	0	0	0
Gaspardone et al. [22] *	prolonged hsp.	43	USS	NA	NA	NA	NA	NA	NA	NA
Rinaldo et al. [23]	Follow-up assessment	111	CT	NM	43	25	NA	NA	NA	NA
Sollini et al. [24]	Follow-up assessment	30	CT, PET	Fibrotic change	6	4	6	0	0	0
Sollini et al. [25]	Follow-up assessment	132	CT, PET	in 4 out of 13 long COVID patients, CT images demonstrated bilateral lung abnormalities—as typically observed in recovered COVID-19 pneumonia—presenting mild [18F]FDG uptake, 2 patients presented moderate/high [18F]FDG uptake in the lung, mediastinal lymph nodes, soft tissue, and breast tissue related to their comorbidities.	6	7	NA	NA	NA	NA
Yin et al. [26]	Follow-up assessment	203	CT	GGO, Consolidation, bronchiectasis, crazy-paving pattern, reticulation	NM	NM	76	7	22	68
Miwa et al. [27]	Follow-up assessment	100	CT	GGO, Consolidation	12	0	0	0	0	12
Total	---	90	CT/PET/USS	---	82	66	85	7	22	85

Abbreviations: FU, follow-up; NM, not mentioned; NA, not applicable. * In this study, Lung Ultra-Sound Score (LUSS) was utilized for mentioning the abnormalities and for the study population the mean score was 7.5. For studies in which two imaging modalities were used, the imaging findings are written here are based on both imaging reports.

**Table 4:** Frequency of lung imaging abnormalities for each different evaluation setting.

Evaluation Setting	Study Design	Positive FU Imaging	Interstitial (Fibrotic) Abnormalities	Pleural Abnormalities	Airway Abnormalities	Other Parenchymal Abnormalities
Follow-up evaluation	Cohort/Case-control/Case-report/Case-series	91/157 (57.9%)	95/167 (56.9%)	7/167 (4.2%)	24/167 (14.4%)	94/167(56.3%)
Re-admission	Case-report/Case-series	10/10 (100%)	3/10 (25.0%)	4/10 (25.0%)	1/10 (8.3%)	8/10 (75.0%)
Prolonged hospitalization	Case-report/Case-series	14/14 (100%)	7/13 (53.8%)	3/13 (23.1%)	3/13 (23.1%)	10/13 (76.9%)

Several studies did not evaluate or report specific imaging findings. Therefore, the total number of patients for each column, in terms of the way of reporting the imaging finding (residual lesions or the specific imaging findings), may differ. The percentages add up to more than 100% because each patient can have more than one finding.

**Table 5:** Frequency of lung imaging abnormalities in cohort/case-control studies that were performed In FU setting.

First Author	Positive FU Imaging	Negative FU Imaging	Category Interstitial (Fibrotic) Abnormalities	Category Pleural Abnormalities	Category Airway Abnormalities	Category Other Parenchymal Abnormalities
Armange et al. [19]	4	19	0	0	0	4
Bai et al. [20]	4	0	3	0	0	1
Cesarone et al. [21]	7	11	7	0	0	0
Rinaldo et al. [23]	43	25	NA	NA	NA	NA
Sollini et al. [24]	6	4	6	0	0	0
Sollin et al. [25]	6	7	NA	NA	NA	NA
Yin et al. [26]	NM	NM	76	0	22	68
Miwa et al. [27]	12	0	0	0	0	12
Total	82/148 (55.4%)	66/148 (44.6%)	92/158 (58.2%)	0/158 (0.0%)	22/158 (14.0%)	85/158 (53.8%)

Footnote: Several studies did not evaluate patients in terms of residual lesions and specific imaging findings. Therefore, there may be inconsistencies in total number of patient counts.

## Data Availability

Not applicable.

## Supplementary Materials

The following supporting information can be downloaded at: https://www.mdpi.com/article/10.3390/jpm13020282/s1, Table S1. Details of included studies; Table S2. Risk factors and comorbidities of long COVID patients in the included articles; Table S3. Frequency of administrated medications during the acute phase in participants of included studies; Table S4. Frequency of long COVID symptoms in participants of included studies; Table S5. Imaging modalities and imaging findings of included case-studies; Table S6. Frequency of lung imaging abnormalities per different evaluation setting. References [49,50,51,52,53,54,55,56,57,58,59,60,61,62,63,64] are cited in the Supplementary Materials
