# Supplementary material for: A Systematic Review of Chest Imaging Findings in Long COVID Patients
Source: J Pers Med. 2023 Feb 1;13(2):282. doi: 10.3390/jpm13020282 (PMC9965323; doi:10.3390/jpm13020282)
Supplement: Supplementary file 1 [file jpm-13-00282-s001.zip › jpm-2172076-Supplementary Materials.pdf]

Supplementary Materials:

Table S1. Details of included studies

First Author	Number of included participants	Age	Female	Male	Long-COVID cases	Age	Female	Male	Long-COVID cases which underwent imaging	Age	Female	Male	ROB	Study design
Armange et al.	214	NM	NM	NM	99	NM	NM	NM	23	44	18	5	fair	cohort
Bai et al.	7	66.3	4	3	4	62.7	2	2	4	62.7	2	2	fair	Case-control
Cesarone et al.	18	56.9	8	10	18	56.9	8	10	18	56.9	8	10	fair	cohort
Gaspardone et al.	70	68	22	48	70	68	22	48	70	68	22	48	fair	cohort
Rinaldo et al.	75	57	32	43	68	NM	NM	NM	68	NM	NM	NM	fair	cohort
Sollini et al.	10	58	3	7	10	58	3	7	10	58	3	7	fair	cohort
Sollini et al.	13	54	5	8	13	53	5	8	13	54	5	8	fair	Case-control
Yin et al	337	53.5	167	170	91	58.7	167	170	91	58.7	34	57	fair	cohort
Miwa et al.	17	61.2	3	14	12	60.2	2	10	12	63.2	2	10	fair	cohort
**Total originals**	761	68.0	244	303	385	59.7	94	147	309	59.7	94	147	fair	---
Tung-Chen et al.[30]	1	41	1	0	1	41	1	0	1	41	1	0	fair	Case-series
Tung-Chen et al.	1	35	1	0	1	35	1	0	1	35	1	0	fair	Case-series
Tung-Chen et al.	1	64	0	1	1	64	0	1	1	64	0	1	fair	Case-series
Scelfo et al.[49]	1	59	1	0	1	59	1	0	1	59	1	0	fair	Case-series
Scelfo et al.	1	59	0	1	1	59	0	1	1	59	0	1	fair	Case-series
Aesif et al.[31]	1	46	0	1	1	46	0	1	1	46	0	1	fair	Case-series
Aesif et al.	1	57	1	0	1	57	1	0	1	57	1	0	fair	Case-series
Aesif et al.	1	57	0	1	1	57	0	1	1	57	0	1	fair	Case-series
Lago et al.[50]	1	72	0	1	1	72	0	1	1	72	0	1	fair	Case-series
Lago et al.	1	59	0	1	1	59	0	1	1	59	0	1	fair	Case-series
Lago et al.	1	63	1	0	1	63	1	0	1	63	1	0	fair	Case-series
Lago et al.	1	62	1	0	1	62	1	0	1	62	1	0	fair	Case-series
Rai et al.[51]	1	84	0	1	1	84	0	1	1	84	0	1	fair	Case-series
Rai et al.	1	65	0	1	1	65	0	1	1	65	0	1	fair	Case-series
Rai et al.	1	36	0	1	1	36	0	1	1	36	0	1	fair	Case-series
Singh et al.[52]	1	45	1	0	1	45	1	0	1	45	1	0	fair	Case-series
**Total case-series**	16	56.5	7	9	16	56.5	7	9	17	56.5	7	10	fair	Case-series
Abdelnour et al.[53]	1	68	1	0	1	68	1	0	1	68	1	0	fair	Case-report
Betelli et al.[54]	1	67	0	1	1	67	0	1	1	67	0	1	fair	Case-report
Horii et al.[55]	1	66	0	1	1	66	0	1	1	66	0	1	fair	Case-report
Zhou et al.[56]	1	40	0	1	1	40	0	1	1	40	0	1	fair	Case-report
Alhiyari et al.[32]	1	60	0	1	1	60	0	1	1	60	0	1	fair	Case-report
Aissaoui et al.[59]	1	61	0	1	1	61	0	1	1	61	0	1	fair	Case-report
Dayco et al.[58]	1	69	0	1	1	69	0	1	1	69	0	1	fair	Case-report
Garg et al.[28]	1	61	0	1	1	61	0	1	1	61	0	1	fair	Case-report
Hamad et al.[33]	1	42	0	1	1	42	0	1	1	42	0	1	fair	Case-report
Heiss et al.[29]	1	60	0	1	1	60	0	1	1	60	0	1	fair	Case-report
Liu et al.[59]	1	57	1	0	1	57	1	0	1	57	1	0	fair	Case-report

Malik et al.[34]	1	67	0	1	1	67	0	1	1	67	0	1	fair	Case-report
Mazzolini et al.[60]	1	74	1	0	1	74	1	0	1	74	1	0	fair	Case-report
Mitrani et al.[61]	1	55	0	1	1	55	0	1	1	55	0	1	fair	Case-report
Susanto et al.[62]	1	46	0	1	1	46	0	1	1	46	0	1	poor	Case-report
Zhu et al.[63]	1	30	0	1	1	30	0	1	1	30	0	1	fair	Case-report
Boehm et al.[64]	1	70	0	1	1	70	0	1	1	70	0	1	fair	Case-report
**Total case-reports**	17	58.4	3	14	17	58.4	3	14	30	57.7	3	27	---	Case-report

Table S2. Risk factors and comorbidities of Long-COVID patients in the included articles

**First Author**	**Previously smoker**	**Currently smoker**	**Hypertension**	**Diabetes**	**Chronic obstructive lung disease**	**Autoimmune Dissease**	**Asthma**	**Chronic polmunary Disease**	**Intubated cases**
Armange et al.	NM	4	NM	NM	NM	NM	4	NM	0
Bai et al.	NM	NM	1	0	0	0	0	0	NM
Cesarone et al.	NM	NM	NM	NM	NM	NM	NM	NM	NM
Gaspardone et al.	NM	5	37	15	4	0	0	0	43
Rinaldo et al.	NM	NM	NM	NM	NM	NM	NM	NM	NM
Sollini et al.	NM	NM	NM	NM	NM	NM	NM	NM	2
Sollini et al.	NM	NM	3	3	0	1	0	0	NM
Yin et al.	NM	8	NM	NM	NM	NM	NM	NM	NM
Miwa et al.	6	2	6	5	0	0	1	0	12
Tung-Chen et al.	NM	NM	NM	NM	NM	NM	NM	NM	NM
Tung-Chen et al.	NM	NM	NM	NM	NM	NM	NM	NM	NM
Tung-Chen et al.	NM	NM	NM	NM	NM	NM	NM	NM	NM
Scelfo et al.	NM	0	0	0	0	1	0	0	0
Scelfo et al.	NM	0	1	0	0	0	0	0	1
Aesif et al.	NM	NM	1	0	0	0	0	0	1
Aesif et al.	NM	NM	0	0	0	0	0	0	0
Aesif et al.	NM	NM	1	0	0	0	0	0	0
Lago et al.	1	0	0	0	0	0	0	0	0
Lago et al.	1	0	1	0	0	0	0	0	0
Lago et al.	1	0	1	0	0	0	0	0	0
Lago et al.	1	0	0	1	0	0	0	0	0
Rai et al.	0	0	0	0	0	0	0	0	0
Rai et al.	0	0	0	0	0	0	0	0	0
Rai et al.	0	0	0	0	0	0	0	0	0
Singh et al.	0	0	1	0	0	0	0	0	0
Abdelnour et al.	NM	NM	0	0	0	0	0	0	1
Betelli et al.	NM	NM	1	0	0	0	0	0	0
Horii et al.	1	1	1	0	0	0	0	0	1
Zhou et al.	NM	NM	0	0	0	0	0	0	0
Alhiyari et al.	0	0	0	1	0	0	0	0	0
Aissaoui et al.	0	0	0	0	0	0	1	0	0
Dayco et al.	NM	NM	0	0	0	0	0	0	0

Garg et al.	*NM*	*NM*	*0*	*1*	*0*	*0*	*0*	*0*	0
Hamad et al.	*NM*	*NM*	*NM*	*NM*	*NM*	*NM*	*NM*	*NM*	NM
Heiss et al.	*NM*	*NM*	*NM*	*NM*	*NM*	*NM*	*NM*	*NM*	0
Liu et al.	*NM*	*NM*	*NM*	*NM*	*NM*	*NM*	*NM*	*NM*	NM
Malik et al.	*NM*	*NM*	*1*	*0*	*0*	*0*	*0*	*0*	0
Mazzolini et al.	*1*	*1*	*0*	*0*	*1*	*0*	*0*	*0*	0
Mitrani et al.	*NM*	*NM*	*NM*	*NM*	*NM*	*NM*	*NM*	*NM*	0
Susanto et al.	*0*	*0*	*0*	*0*	*0*	*0*	*0*	*0*	NM
Zhu et al.	*0*	*0*	*0*	*0*	*0*	*0*	*0*	*0*	0
Boehm et al.	*1*	*0*	*1*	*0*	*0*	*0*	*0*	*0*	1

Table S3. Frequency of administrated medications during the acute phase in participants of included studies

First Author	***Cases***	***COVID Medical Treatment***	***Anti-coagulants***	***Hydroxychloroquine***	***Corticostroids***	***Oral Antibiotics***	***Intra-venous Antibiotics***	***Antiviral therapy***	***IL-1 inhibitor***	***IL-6 inhibitors***	***Immunoglo therapy***
Armange et al.	*23*	*NM*	*NM*	*NM*	*NM*	*NM*	*NM*	*NM*	*NM*	*NM*	*NM*
Bai et al.	*4*	*4*	*NM*	*0*	*3*	*3*	*0*	*4*	*0*	*0*	*0*
Cesarone et al.	*18*	*NM*	*NM*	*NM*	*NM*	*NM*	*NM*	*NM*	*NM*	*NM*	*NM*
Gaspardone et al.	*70*	*55*	*NM*	*48*	*NM*	*25*	*NM*	*39*	*18*	*7*	*NM*
Rinaldo et al.	*68*	*NM*	*NM*	*NM*	*NM*	*NM*	*NM*	*NM*	*NM*	*NM*	*NM*
Sollini et al.	*10*	*NM*	*3*	*3*	*NM*	*NM*	*NM*	*NM*	*NM*	*NM*	*NM*
Sollini et al.	*13*	*3*	*7*	*6*	*5*	*4*	*5*	*4*	*0*	*0*	*0*
Yin et al.	*91*	*NM*	*NM*	*NM*	*NM*	*NM*	*NM*	*83*	*NM*	*NM*	*55*
Miwa et al.	*12*	*NM*	*NM*	*NM*	*NM*	*NM*	*NM*	*NM*	*NM*	*NM*	*NM*
Tung-Chen et al.	*1*	*NM*	*NM*	*NM*	*NM*	*NM*	*NM*	*NM*	*NM*	*NM*	*NM*
Tung-Chen et al.	*1*	*NM*	*NM*	*NM*	*NM*	*NM*	*NM*	*NM*	*NM*	*NM*	*NM*
Tung-Chen et al.	*1*	*NM*	*NM*	*NM*	*NM*	*NM*	*NM*	*NM*	*NM*	*NM*	*NM*
Scelfo et al.	*1*	*1*	*0*	*1*	*0*	*0*	*0*	*1*	*0*	*1*	*0*
Scelfo et al.	*1*	*1*	*0*	*1*	*0*	*0*	*0*	*1*	*0*	*1*	*0*
Aesif et al.	*1*	*1*	*0*	*0*	*0*	*0*	*0*	*1*	*0*	*1*	*0*
Aesif et al.	*1*	*1*	*0*	*0*	*0*	*0*	*0*	*1*	*0*	*0*	*0*
Aesif et al.	*1*	*1*	*1*	*1*	*1*	*1*	*0*	*0*	*1*	*1*	*0*
Lago et al.	*1*	*NM*	*NM*	*NM*	*NM*	*NM*	*NM*	*NM*	*NM*	*NM*	*NM*
Lago et al.	*1*	*NM*	*NM*	*NM*	*NM*	*NM*	*NM*	*NM*	*NM*	*NM*	*NM*
Lago et al.	*1*	*NM*	*NM*	*NM*	*NM*	*NM*	*NM*	*NM*	*NM*	*NM*	*NM*
Lago et al.	*1*	*NM*	*NM*	*NM*	*NM*	*NM*	*NM*	*NM*	*NM*	*NM*	*NM*
Rai et al.	*1*	*1*	*1*	*0*	*1*	*0*	*0*	*1*	*0*	*0*	*0*
Rai et al.	*1*	*1*	*1*	*0*	*1*	*0*	*0*	*0*	*0*	*0*	*0*
Rai et al.	*1*	*1*	*0*	*0*	*1*	*0*	*1*	*1*	*0*	*0*	*0*
Singh et al.	*1*	*1*	*1*	*0*	*1*	*1*	*0*	*0*	*0*	*0*	*0*
Abdelnour et al.	*1*	*0*	*0*	*0*	*0*	*0*	*1*	*0*	*0*	*0*	*0*
Betelli et al.	*1*	*1*	*1*	*1*	*0*	*1*	*1*	*0*	*0*	*0*	*0*

Horii et al.	*1*	*1*	*0*	*1*	*0*	*0*	*1*	*1*	*0*	*1*	*0*
Zhou et al.	*1*	*1*	*0*	*0*	*1*	*0*	*0*	*1*	*0*	*0*	*1*
Alhiyari et al.	*1*	*1*	*0*	*0*	*1*	*0*	*1*	*1*	*0*	*0*	*0*
Aissaoui et al.	*1*	*0*	*1*	*0*	*1*	*0*	*0*	*0*	*0*	*0*	*0*
Dayco et al.	*1*	*1*	*0*	*0*	*1*	*0*	*0*	*0*	*0*	*0*	*0*
Garg et al.	*1*	*1*	*0*	*0*	*1*	*0*	*0*	*1*	*0*	*0*	*0*
Hamad et al.	*1*	*0*	*0*	*0*	*0*	*0*	*0*	*0*	*0*	*0*	*0*
Heiss et al.	*1*	*0*	*0*	*0*	*0*	*0*	*0*	*0*	*0*	*0*	*0*
Liu et al.	*1*	*1*	*0*	*0*	*1*	*0*	*0*	*1*	*0*	*0*	*0*
Malik et al.	*1*	*1*	*1*	*0*	*1*	*1*	*1*	*1*	*0*	*0*	*0*
Mazzolini et al.	*1*	*1*	*1*	*1*	*1*	*1*	*0*	*0*	*0*	*1*	*0*
Mitrani et al.	*1*	*1*	*0*	*0*	*1*	*0*	*1*	*0*	*0*	*0*	*0*
Susanto et al.	*1*	*1*	*0*	*1*	*0*	*1*	*1*	*1*	*0*	*0*	*0*
Zhu et al.	*1*	*0*	*0*	*0*	*1*	*0*	*1*	*1*	*0*	*0*	*0*
Boehm et al.	*1*	*1*	*0*	*1*	*1*	*1*	*1*	*1*	*0*	*0*	*0*

Table S4. Frequency of Long-COVID symptoms in participants of included studies

First Author	**Cough**	**Fatigue**	**Weakness**	**Dyspnea**	**Dyspnea on excertin**
Armange et al.	NM	NM	9	19	NM
Bai et al.	4	1	0	0	0
Cesarone et al.	2	5	NM	6	NM
Gaspardone et al.	NM	NM	NM	NM	NM
Rinaldo et al.	NM	NM	NM	NM	NM
Sollini et al.	NM	7	NM	7	NM
Sollini et al.	0	8	0	9	0
Yin et al.	NM	NM	NM	91	NM
Miwa et al.	NM	NM	NM	NM	7
Tung-Chen et al.	0	0	1	0	1
Tung-Chen et al.	0	0	1	0	1
Tung-Chen et al.	0	0	1	0	1
Scelfo et al.	0	1	0	1	0
Scelfo et al.	0	0	1	1	0
Aesif et al.	NM	NM	NM	NM	NM
Aesif et al.	NM	NM	NM	NM	NM
Aesif et al.	NM	NM	NM	NM	NM
Lago et al.	NM	1	NM	0	NM
Lago et al.	NM	1	NM	0	NM
Lago et al.	NM	1	NM	0	NM
Lago et al.	NM	0	NM	1	NM
Rai et al.	NM	NM	NM	NM	NM
Rai et al.	NM	NM	NM	NM	1
Rai et al.	NM	NM	NM	NM	1
Singh et al.	0	NM	NM	1	NM

Abdelnour et al.	NM	NM	NM	NM	NM
Betelli et al.	0	0	0	1	0
Horii et al.	NM	NM	NM	NM	NM
Zhou et al.	NM	NM	NM	NM	NM
Alhiyari et al.	1	NM	NM	NM	NM
Aissaoui et al.	0	0	0	1	1
Dayco et al.	NM	NM	NM	NM	NM
Garg et al.	NM	NM	NM	NM	1
Hamad et al.	1	0	0	1	NM
Heiss et al.	NM	NM	NM	NM	1
Liu et al.	1	NM	NM	NM	NM
Malik et al.	NM	NM	NM	1	NM
Mazzolini et al.	NM	NM	NM	1	NM
Mitrani et al.	NM	NM	NM	1	1
Susanto et al.	NM	NM	NM	1	NM
Zhu et al.	1	NM	NM	1	NM
Boehm et al.	NM	NM	NM	NM	NM

Table S5. Imaging modalities and imaging findings of included case-studies

First Author	***Evaluation setting***	***Evaluation time in days (performing the imaging)***	***Imaging Modality***	***imaging finding***	***Category Interstitial***	***Category Pleural***	***Category Airway***	***Category Parenchymal***
Tung-Chen et al.[30]	*Re-admission*	*56*	*CT, US*	*pleural thickening*	*0*	*1*	*0*	*0*
Tung-Chen et al.	*Re-admission*	*56*	*CT, US*	*GGO*	*0*	*0*	*0*	*1*
Tung-Chen et al.	*Re-admission*	*56*	*CT, US*	*Fibrotic change*	*0*	*0*	*0*	*0*
Scelfo et al.[49]	*Re-admission*	*29*	*CT*	*GGO, Consolidation, linear scaring, interstitial thickening*	*1*	*0*	*0*	*1*
Scelfo et al.	*Re-admission*	*28*	*CT*	*Consolidation, pleural effusion, reticulation*	*1*	*1*	*0*	*1*
Aesif et al.[31]	*Prolonged hospitalization*	*38*	*CT*	*GGO, Consolidation, pneumothorax, hemopneumothorax, airspace opacity*	*0*	*1*	*0*	*1*
Aesif et al.	*Prolonged hospitalization*	*40*	*CT*	*Consolidation, pleural effusion, airspace opacity*	*0*	*1*	*0*	*1*
Aesif et al.	*Prolonged hospitalization*	*122*	*CXR*	*complete opacification, volume loss*	*0*	*0*	*0*	*1*
Lago et al.[50]	*Follow-up assessment*	*90*	*CT*	*GGO, reticular opacity, volume loss*	*0*	*0*	*0*	*1*
Lago et al.	*Follow-up assessment*	*90*	*CT*	*GGO, reticular opacity, volume loss*	*0*	*0*	*0*	*1*
Lago et al.	*Follow-up assessment*	*90*	*CT*	*GGO, reticular opacity, volume loss*	*0*	*0*	*0*	*1*
Lago et al.	*Follow-up assessment*	*90*	*CT*	*GGO, reticular opacity, volume loss*	*0*	*0*	*0*	*1*
Rai et al.[51]	*Prolonged hospitalization*	*28*	*CT*	*reticular opacity, bronchiolectasis, bronchiectasis, emphysematous changes, architectural distortion, honeycomb-like appearance*	*1*	*0*	*1*	*1*

Rai et al.	*Prolonged hospitalization*	*42*	*CT, CXR*	*GGO, Consolidation, reticulation, honeycomb-like appearance, volume loss*	*1*	*0*	*0*	*1*
Rai et al.	*prolonged hospitalization*	*29*	*CT*	*GGO, Fibrotic change*	*1*	*0*	*0*	*1*
Singh et al.[52]	*prolonged hospitalization*	*31*	*CT*	*GGO, bronchiectasis, interstitial thickening*	*1*	*0*	*1*	*0*
Abdelnour et al.[53]	*prolonged hospitalization*	*41*	*CXR*	*Aeriation*	*0*	*0*	*0*	*1*
Betelli et al.[54]	*re-admission*	*35*	*CT*	*GGO, Consolidation, pleural effusion*	*0*	*1*	*0*	*1*
Horii et al.[55]	*prolonged hsp*	*50*	*CT*	*GGO, Consolidation, pleural effusion, atelectasis*	*0*	*1*	*0*	*1*
Zhou et al.[56]	*re-admission*	*44*	*CT*	*Consolidation*	*0*	*0*	*0*	*1*
Alhiyari et al.[32]	*re-admission*	*130*	*CT*	*GGO, bronchiectasis, interlobular septal thickening, reticulation, honeycomb-like appearance, interstitial pneumonia pattern*	*1*	*0*	*1*	*1*
Aissaoui et al.[59]	*Follow-up assessment*	*150*	*CT*	*GGO, reticulation, Fibrotic change, emphysematous changes*	*1*	*0*	*0*	*1*
Dayco et al.[58]	*prolonged hospitalization*	*90*	*CT*	*Fibrotic change, pulmonary edema*	*1*	*0*	*0*	*0*
Garg et al.[28]	*Follow-up assessment*	*53*	*CT*	*interlobular septal thickening, parenchymal band, Fibrotic change, bronchiectasis, bronchiolectasis, crazy-paving pattern*	*1*	*0*	*1*	*1*
Hamad et al.[33]	*re-admission*	*35*	*CXR*	*pneumothorax, airspace opacity*	*0*	*1*	*0*	*1*
Heiss et al.[29]	*Follow-up assessment*	*97*	*CT*	*GGO, Consolidation*	*0*	*0*	*0*	*1*
Liu et al.[59]	*Re-admission*	*46*	*CT*	*GGO, Consolidation*	*0*	*0*	*0*	*1*
Malik et al.[34]	*prolonged hospitalization*	*42*	*CT*	*GGO, Fibrotic change, bronchiectasis, architectural distortion, linear scaring, airspace disease, COVID pneumonia*	*1*	*0*	*1*	*1*
Mazzolini et al.[60]	*prolonged hospitalization*	*55*	*CT*	*GGO, Fibrotic change, architectural distortion, emphysematous changes, volume loss*	*1*	*0*	*0*	*0*
Mitrani et al.[61]	*Follow-up assessment*	*60*	*CXR*	*bilateral pneumonia/viral pneumonitis*	*0*	*0*	*0*	*1*
Susanto et al.[62]	*Follow-up assessment*	*75*	*CT*	*Fibrotic change, Consolidation, bronchiolectasis, parenchymal band*	*1*	*0*	*1*	*1*
Zhu et al.[63]	*prolonged hospitalization*	*28*	*CT*	*Fibrotic change*	*NM*	*NM*	*NM*	*NM*
Boehm et al.[64]	*prolonged hospitalization*	*40*	*CT*	*Consolidation*	*NM*	*NM*	*NM*	*1*
Total	*---*	*60.18*	*CT/CXR/US*	*---*	*13*	*7*	*20*	*27*

Table S6. Frequency of lung imaging abnormalities per different evaluation setting

Evaluation setting	*First Author*	*Number of Long COVID cases underwent imaging*	*Positive imaging*	*Negative imaging*	*Category Interstitial abnormalities*	*Category Pleural abnormalities*	*Category Airway abnormalities*	*Category Parenchymal abnormalities*
Follow-up evaluation	*Armange et al.*	*23*	*4*	*19*	*0*	*0*	*0*	*4*
	Bai et al.	*4*	*4*	*0*	*3*	*0*	*0*	*1*
	Cesarone et al.	*18*	*7*	*11*	*7*	*0*	*0*	*0*
	Rinaldo et al.	*68*	*43*	*25*	*NA*	*NA*	*NA*	*NA*
	Sollini et al.	*10*	*2*	*8*	*6*	*0*	*0*	*0*
	Sollini et al.	*13*	*4*	*9*	*NA*	*NA*	*NA*	*NA*
	Yin et al.	*91*	*NM*	*NM*	*76*	*7*	*22*	*68*
	Miwa et al.	*12*	*12*	*0*	*0*	*0*	*0*	*12*
	Lago et al.	*1*	*1*	*0*	*0*	*0*	*0*	*1*
	Lago et al.	*1*	*1*	*0*	*0*	*0*	*0*	*1*
	Lago et al.	*1*	*1*	*0*	*0*	*0*	*0*	*1*
	Lago et al.	*1*	*1*	*0*	*0*	*0*	*0*	*1*
	Aissaoui et al.	*1*	*1*	*0*	*1*	*0*	*0*	*1*
	Garg et al.	*1*	*1*	*0*	*1*	*0*	*1*	*1*
	Heiss et al.	*1*	*1*	*0*	*0*	*0*	*0*	*1*
	Mitrani et al.	*1*	*1*	*0*	*0*	*0*	*0*	*1*
	Susanto et al.	*1*	*1*	*0*	*1*	*0*	*1*	*1*
	Total FU evaluation	*248*	*85*	*72*	*95*	*7*	*24*	*94*
Re-admission	*Tung-Chen et al.*	*1*	*1*	*0*	*0*	*1*	*0*	*0*
	Tung-Chen et al.	*1*	*1*	*0*	*0*	*0*	*0*	*1*
	Tung-Chen et al.	*1*	*1*	*0*	*0*	*0*	*0*	*0*
	Scelfo et al.	*1*	*1*	*0*	*1*	*0*	*0*	*1*
	Scelfo et al.	*1*	*1*	*0*	*1*	*1*	*0*	*1*
	Betelli et al.	*1*	*1*	*0*	*0*	*1*	*0*	*1*
	Zhou et al.	*1*	*1*	*0*	*0*	*0*	*0*	*1*
	Alhiyari et al.	*1*	*1*	*0*	*1*	*0*	*1*	*1*
	Hamad et al.	*1*	*1*	*0*	*0*	*1*	*0*	*1*
	Liu et al.	*1*	*1*	*0*	*0*	*0*	*0*	*1*
	Total Re-admission	*10*	*10*	*0*	*3*	*3*	*1*	*9*
Prolonged hospitalization	Gaspardone et al.	*70*	*NM*	*NM*	*NA*	*NA*	*NA*	*NA*
	Aesif et al.	*1*	*1*	*0*	*0*	*1*	*0*	*1*
	Aesif et al.	*1*	*1*	*0*	*0*	*1*	*0*	*1*
	Aesif et al.	*1*	*1*	*0*	*0*	*0*	*0*	*1*
	Rai et al.	*1*	*1*	*0*	*1*	*0*	*1*	*1*
	Rai et al.	*1*	*1*	*0*	*1*	*0*	*0*	*1*
	Rai et al.	*1*	*1*	*0*	*1*	*0*	*0*	*1*
	Singh et al.	*1*	*1*	*0*	*1*	*0*	*1*	*0*

	Abdelnour et al.	*1*	*1*	*0*	*0*	*0*	*0*	*1*
	Horii et al.	*1*	*1*	*0*	*0*	*1*	*0*	*1*
	Dayco et al.	*1*	*1*	*0*	*1*	*0*	*0*	*0*
	Malik et al.	*1*	*1*	*0*	*1*	*0*	*1*	*1*
	Mazzolini et al.	*1*	*1*	*0*	*1*	*0*	*0*	*0*
	Zhu et al.	*1*	*NM*	*NM*	*NM*	*NM*	*NM*	*NM*
	Boehm et al.	*1*	*NM*	*NM*	*NM*	*NM*	*NM*	*1*
	Total prolonged hospitalization	*84*	*12*	*0*	*7*	*3*	*3*	*10*
